# Spatio-Temporal Analysis and Influencing Factors of Rural Resilience from the Perspective of Sustainable Rural Development

**DOI:** 10.3390/ijerph191912294

**Published:** 2022-09-27

**Authors:** Mei Yang, Mengyun Jiao, Jinyu Zhang

**Affiliations:** 1 School of Management, Chongqing University of Technology, Chongqing 400054, China; 2 Collaborative Research Center for Innovation-Driven Entrepreneurship, Chongqing University of Technology, Chongqing 400054, China

**Keywords:** sustainable rural development, rural revitalization, rural resilience, spatial and temporal evolution, configuration analysis

## Abstract

Rural resilience is not only a comprehensive reflection of “thriving businesses, pleasant living environments, social etiquette and civility, effective governance, and prosperity”. It is also the unity of resilience in industry, ecology, culture, organization and livelihood. This paper uses the entropy weight-TOPSIS method to measure the rural resilience level in 31 regions in China and analyzes the configuration of influencing factors with the Fuzzy-set qualitative comparative analysis (fsQCA). The results of the study are as follows: (1) The level of rural resilience in China showed a stable increase from 2010 to 2019, but the overall level was low, with large regional disparities, showing a significant positive spatial correlation. (2) In the high-level rural resilience explanatory path, labor-driven, cultural-driven and market–labor–technology linkage-driven play a core role, while administrative force is not playing a significant role. In the explanation path of non-high level rural resilience, the market–labor absent, administrative–market absent and cultural absent hinder the improvement of rural resilience. In summary, we put forward the following suggestions. Policy renovation and support should be strengthened. Adaption to local conditions should be considered in order to achieve sustainable and differentiated development. Development should be coordinated and balanced in different regions so as to achieve an overall resilience level in rural areas.

## 1. Introduction

Due to the COVID-19 pandemic, farmers’ livelihood, the transformation of agriculture and sustainable rural development have been significantly affected. The question of how to achieve sustainable development in a challenging environment has attracted attention worldwide. The world environment is full of turbulence, and fluctuations in agriculture pose enormous challenges for farmers, countries and the world. China is one of the world’s largest agricultural countries. In 2020, China’s rural population accounted for 36.1% of the total population. Therefore, China is facing problems in keeping farmers’ livelihood and income. Though with the rapid urbanization pace, China’s rural development still faces obstacles caused by production mode, disparities in farmers’ income and employment and outdated concepts. In light of this situation, the “Rural Revitalization Strategy” was proposed in the report of the 19th National Congress of CPC on 18 October 2017 to solve agricultural, rural and peasant problems. The rural revitalization strategy was proposed in the report of the 19th National Congress of the Communist Party of China on 18 October 2017. The strategy promotes high-quality rural development. The strategy aims to achieve thriving businesses, pleasant living environments, social etiquette and civility, effective governance and prosperity through the revitalization of industry, talent cultivation, culture, ecology and organization. At the same time, this strategy is a long-term policy of resilient and sustainable development based on the ability of rural areas in line with the reality and development law of rural areas [1]. The world is currently experiencing climate change, frequent natural environmental disasters, the impact of COVID-19, the livelihood of rural farmers and unbalanced urban–rural development. In this context, it is necessary to enrich rural resilience and enhance the characteristics of rural maintenance and development. Mitigating disturbances and preventing and resolving risks are important supports for promoting rural construction and achieving high-quality rural development and rural revitalization.

Resilience is derived from the Latin word “resilio”, which means “to return to the original state”, and its related research has gone through three stages: engineering resilience, ecological resilience and evolutionary resilience [2]. Engineering resilience refers to the ability of physical properties of materials, surfaces, structures, or systems to withstand shocks without deformation [3], which is the restoration of initial homeostasis. Ecological resilience was first proposed by Canadian ecologist Holling, stressing resilience as the ability of ecosystems to absorb changes and to sustain and restore equilibrium [4], thus shaping new homeostasis. Evolutionary resilience emerged in the 1990s, which regards resilience as a dynamic system property that is adaptive, learning and innovative and a capacity for change, adaptation and transformation inspired by complex social–ecological systems in response to pressures and constraints [5]. Within the framework of evolutionary resilience, discussions related to how communities and societies respond to changes and risks in the social environment have become a hot topic of research in the academic community [6,7]. Adger (2000) argues that social resilience is defined as the ability of groups or communities to cope with external pressures and disruptions due to social, political and environmental changes [8]. Norris et al. (2008) defined community resilience as a process that links a range of adaptive capacities to post-disturbance functioning and positive trajectories of adaptation [9].

The rural area is a territorial complex with natural, social and economic characteristics, combining multiple functions such as production, living, ecology and culture. It promotes each other and coexists with cities and towns, forming the main space for human activities. While focusing on urban resilience, rural resilience has also received academic attention and has gradually become a research hotspot. Heijman defines rural resilience as the ability of rural areas to adapt to a changing external environment so as to maintain a satisfactory standard of living [10]. Li argues that a resilient rural community has the ability to cope with unwelcome challenges and adapt to a changing external environment by forming a higher standard of living [11]. The existing literature mainly focuses on rural resilience in rural economy [12], agricultural land [13,14], disaster management [15,16], farm household livelihood [17] and community development [18]. The construction of a rural resilience indicator system has been explored in a multidimensional and multidisciplinary manner using field research and questionnaires [19,20], entropy weight method and approximating ideal solution ranking (TOPSIS) [21], but a unified measurement system has not yet been developed. For example, Huang developed a resilience assessment index system including four types of resilience: engineering, ecological, economic and social, to assess the changes in rural resilience by withdrawal mechanism for rural homesteads (WMRH) policy [22]. Based on the analysis of the rural system resilience (RRS) mechanism, Li and Jin constructed a village-level resilience assessment framework based on three dimensions, resources, form and function [19]. Wilson combined social, cultural, natural, economic and political factors to assess the resilience level of rural communities [18]. While Wang and Dai constructed a system of indicators for measuring the resilience of rural habitat systems from five major subsystems: natural subsystem, human subsystem, housing subsystem, supporting subsystem and social subsystem in the rural habitat environment [23]. Regarding the study of factors influencing rural resilience, McManus emphasized the importance of economic and employment conditions in maintaining the resilience of local rural communities [24]. Quaranta showed through pilot experiments that high natural social–economic diversity in a given socio-ecological system promotes high-quality rural development levels [25]. Rahmawati suggested that land degradation problems can negatively affect environmental, social and economic conditions, thus reducing rural resilience [14]. Hanson found that rural ICTs have a positive impact on rural resilience [26]. Meanwhile, some scholars proved that rural culture [27], public sense of belonging [28] and government policies [16] could have a significant impact on rural resilience. Although the above-mentioned studies are enlightening, they mostly refer to the framework of urban resilience assessment and construct the rural resilience index system mainly from economic, social, infrastructure and ecological dimensions and are not closely related to the rural revitalization strategy. In addition, the resilience manifestation of rural areas under the change in the internal and external environment is the inevitable result of the joint action of some specific key elements. The existing literature often uses the obstacle degree model [14], GeogDetector-based model [29], regression analysis [24] and other methods to explore the linear relationship among the influencing factors of rural resilience, mainly focusing on the independent effects of different factors. There are some limitations in the lack of configuration effect analysis of multiple influencing factors on rural resilience and the influence mechanism research on complex systems. The rural revitalization strategy is a new requirement for China’s economic and social development [30], as well as an important policy document for China’s rural construction. Therefore, based on the differences in resilience between urban and rural areas, this paper innovatively integrates the connotation, requirements and planning of the rural revitalization strategy into the measurement of rural resilience. We took the general requirements of the rural revitalization of “ thriving businesses, pleasant living environments, social etiquette and civility, effective governance, and prosperity” as the dimension source of rural resilience. We did not construct rural resilience on the urban resilience indicator system but combined it with a rural resilience strategy. We constructed rural resilience from five aspects: industrial resilience, ecological resilience, cultural resilience, organizational resilience and livelihood resilience, which is an important innovation of this paper. At the same time, we adopted the Fuzzy Set Qualitative Comparative Analysis (fsQCA) method to explore the influence of administrative force, market force, labor force, technology force and cultural force on rural resilience. The five factors of administrative force, market force, labor force, technology force and cultural force were used to explore the conditional configuration and nonlinear effects of these factors on rural resilience and to elucidate the complex driving mechanism of different influencing factors on rural resilience. This is also an important innovation of this paper. By clarifying China’s research on rural resilience, we hope to provide a new way to solve the current rural development problems in the world. We constructed an indicator system on the basis of China’s practice with the aim of providing new perspectives and references for the study of rural resilience in other countries through China’s experience.

## 2. Materials and Methods

### 2.1. Research Framework

**Figure 1:**
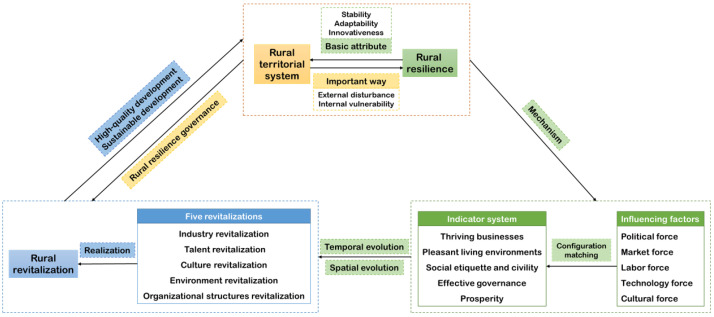
Research framework in rural resilience under rural revitalization strategy.

In contrast to the rapid development of the market economy and urbanization, the countryside is underdeveloped, disadvantaged and marginalized, and the gap between urban and rural areas is too large [1]. In order to achieve the comprehensive revitalization of the countryside, we must aim at solving the practical problem in the countryside with a focus on issues concerning agriculture, the countryside and farmers [31]. The object of rural revitalization is the rural territorial system, which is a systematic process of reorganizing, reconstructing and upgrading the elements of the rural system [32]. As a basic attribute of a rural system, rural resilience not only focuses on the regional characteristics of the rural system [33] but also explains the complexity of multiple elements and multi-functions of the rural system and fully reflects the dynamics and evolution of the rural system. Based on this, we constructed a research framework of rural resilience under the guidance of a rural revitalization strategy (Figure 1).

#### 2.1.1. Theoretical Logic of Rural Territorial System, Rural Revitalization Strategy and Rural Resilience

The rural territorial system is a rural spatial system with a specific structure, function and inter-regional connection composed of cultural, economic and environmental elements [34]. Rural resilience is the basic attribute of the rural territorial system. In the process of system operation, according to the external disturbance and internal vulnerability of the system, the configuration of elements in the system is optimized so that the level of rural resilience evolves and varies. With the gradual improvement of resilience, the rural territorial system adjusts its structure and functions according to internal and external shocks and achieves new and resilient development. Rural resilience contributes to the stability, adaptability and innovation of rural territorial systems. It can promote coordinated development in the economy, environment, society, resources and culture. Rural resilience can also absorb external disturbances to adjust from the original system equilibrium to the new equilibrium to maintain adaptability and cultivate the innovation of the system by means of learning.

The internal mechanisms of rural resilience and rural revitalization are highly consistent and synergistic, and the rural revitalization strategy contains rich concepts of resilience. In the process of implementing the rural revitalization strategy, on the one hand, China faces the challenge of unstable agricultural trade in the international market, and on the other hand, the country is in a critical period of domestic rural reform and transformation. Therefore, China needs to enhance the overall strength of rural resilience to cope properly with international and domestic risks and challenges [35]. Vulnerability analysis based on rural resilience helps rural areas to cope with and recover from problems in infrastructure, resources and culture, which is highly compatible with the general requirements of rural revitalization strategy (Appendix A, Item 1).

Therefore, starting from the basic attribute of rural resilience, we conducted in-depth research on the level of rural resilience in China. It is of great significance to promote high-quality and sustainable rural development by strengthening the ability of rural systems to cope with risks and changes [36]. It is not difficult to find that the sustainable development of rural areas, the enhancement of rural resilience and the realization of rural revitalization goals are all dynamic, and it is a long-term evolutionary process. Through the panel data, we mined the spatial information and temporal characteristics and conducted a statistical analysis of spatio-temporal data on rural resilience. Based on the scientific cognition of rural revitalization strategy, we discussed the temporal–spatial distribution and driving path of rural resilience. By studying the spatial and temporal distribution of rural resilience and the configuration analysis of influencing factors, we can fully explain the performance of short-term shocks and the characteristics of long-term changes in rural resilience. Under the guidance of the rural revitalization strategy, China could realize industry revitalization, talent revitalization, culture revitalization, environment revitalization and organizational structures revitalization by improving rural resilience (Appendix A, Item 2).

#### 2.1.2. Constructing Rural Resilience Evaluation System

At present, scholars mostly refer to the research framework of urban resilience and construct the evaluation index system of rural resilience from economic, social, infrastructural and ecological dimensions [11,18,36]. However, there are significant differences between rural and urban areas in many aspects such as population, space, economy and society. Correspondingly, the concept and technology of coping with disasters, risks and disturbances in rural areas are different from those in cities. Therefore, the evaluation system of rural resilience needs to form a unique framework and characteristics. We combined resilience theory with Chinese practice based on the holistic, open and dynamic nature of the rural territorial system. Based on the general requirements of rural revitalization strategy(Appendix A, Item 1), we built a rural resilience evaluation system of industrial resilience, ecological resilience, cultural resilience, organizational resilience and livelihood resilience. Then, we built the rural resilience evaluation system for industrial resilience, ecological resilience, cultural resilience, organizational resilience and livelihood resilience. Meanwhile, the indexes were selected with reference to the existing studies [37,38,39,40], and 20 indexes were selected in this study to build the rural resilience evaluation index system (Table 1).

Industrial resilience is the key to rural resilience and is fundamental to the issues concerning agriculture, rural areas and farmers. The rural economy is an important part of the modern economic system. Industrial resilience is the ability of the rural economy to maintain the stability of the economic environment and industrial structure when it is subject to internal and external pressure and impact and is also the ability to realize industry revitalization by adjusting the resource allocation method and agricultural structure of the rural market. In this dimension, four key factors were selected as the characterization indicators of thriving businesses (Table 1).

Ecological resilience is an advantage in enhancing rural resilience and is an important initiative for rural construction. Lucid waters and lush mountains are invaluable assets. Ecological resilience is the ability to resist and resolve the rural ecological environment after it suffers from natural disasters and social pollution. By focusing on natural resilience, we were able to advance the improvement of the rural living environment and thus achieve rural ecological revitalization. In this dimension, four key factors were selected as the characterization indicators of pleasant living environments (Table 1).

Cultural resilience is the soul of enhancing rural resilience and the source of spiritual power for rural development. Culture is the soul of a country or a nation, important support for rural cohesion and creativity and soft power for rural construction and high-quality development. Cultural resilience is the ability of the rural system to respond actively and achieve value reconstruction when the countryside is subject to social changes and external cultural impacts. In this dimension, four key factors are selected as the characterization indicators of social etiquette and civility (Table 1).

Organizational resilience is a guarantee to enhance rural resilience and is an important foundation of rural governance. Rural governance is an important part of China’s national governance system. Organizational resilience is the degree of tolerance and recovery ability shown by local governments and rural organizations in adversity. By standardizing rural leadership institutions, implementing government investment in rural people and finances, and ensuring rural infrastructure construction, rural organizational revitalization can be achieved. In this dimension, four key factors were selected as the characterization indicators of effective governance (Table 1).

Livelihood resilience is fundamental to enhancing rural resilience and is an inevitable choice for the common prosperity of all people. By enabling rural residents to have a sustainable and stable saving capacity and source of income [41], it can free the farmer from worries about food and clothing and ensure them a convenient life. Livelihood resilience requires improved production and living standards for rural residents to be able to recover quickly and respond to disasters. In this dimension, four key factors were selected as the characterization indicators of prosperity (Table 1).

#### 2.1.3. Determining Impact Factors of Rural Resilience

The rural territorial system has the characteristics of multi-scale, multi-level and multi-type [42]. The rural environment is complex and volatile, and all kinds of uncertainty disturbances restrict the high-quality and sustainable development of rural areas. Therefore, there are complex causes and possibilities for rural resilience. Neither the traditional single influencing factor analysis nor linear-logic-based multivariate regression analysis alone can fully explain the changes in rural resilience. Accordingly, we selected a research method that not only explores the problem of multidimensional and multivariate causal complexity [43] but also is advantageous in both case-oriented and variable-oriented approaches [44], namely qualitative comparative analysis (QCA). Through QCA, the influencing factors leading to the change in rural resilience levels were analyzed as a whole. By relying on configuration matching, the configuration of several key factors leading to high or not high rural resilience levels was determined. These configurations represent multiple equivalent paths leading to the results and promote the rural resilience level to provide diversified improvement paths according to the differences of rural territorial systems [45,46]. By referring to the existing literature, this study took the administration to help agriculture, the market to benefit agriculture, talents to strengthen agriculture, science and technology to promote agriculture and culture to enrich agriculture as the entry point. We not only considered the availability of relevant data indicators but also considered the selection and definition of condition variables from five aspects of administrative force, market force, labor force, technology force and cultural force [47,48] (Table 2).

Administrative force reflects the attention and support of governments at all levels to rural construction. It is mainly manifested in the government’s provision of policy inclination and financial support in rural construction. The administrative force is an important driving force to promote the comprehensive development of the rural economy and society and improve rural resilience. Therefore, we selected the per capita finance expenditure of agriculture, forestry and water resources as the specific indicator to reflect the administrative force.

Market force reflects the effective allocation ability of rural market resources, which is closely related to industrial resilience. The higher the market force, the more active the flow of resources, industries and talents in the rural market. Therefore, we selected rural construction input as the specific index to reflect the market force.

The labor force reflects the ability of talent to support rural construction. The labor force has an important impact on rural industrial resilience, ecological resilience and other dimensions [49]. Without a labor force, rural industry and capital investment cannot be transformed into productivity and cannot fully produce economic benefits. Therefore, we selected years of schooling per capita as a specific indicator reflecting the labor force.

Technology force reflects the ability of rural industries, especially agricultural science and technology innovation. Technology provides support for agricultural modernization. Breakthroughs were made in the fields of food security, land protection and seed industry improvement. The basis of agricultural modernization is agricultural mechanization. Modern science and technology, advanced machinery and equipment are needed to facilitate agriculture. Therefore, we selected the level of agricultural machinery as a specific indicator reflecting scientific and technology forces. 

Cultural force reflects the identity and cohesiveness of the countryside based on culture [39]. Culture activities could make villages more cohesive and centripetal and could enhance residents’ sense of identity and belonging to their own place. At the same time, the cultural force can promote the development of rural cultural industries. It transforms rural cultural resources with distinctive regional and ethnic characteristics into cultural productivity. It can realize the deep integration of culture with agriculture, tourism and other industries. Therefore, we selected per capita cultural and entertainment expenditure as a specific indicator to reflect cultural force.

### 2.2. Methods and Data Sources

#### 2.2.1. Entropy-TOPSIS Method

Regarding the evaluation methods of rural resilience, the more commonly used methods are the entropy method, grey evaluation method, analytic hierarchy process, entropy-TOPSIS method, etc. In order to avoid too many subjective factors that could interfere with the evaluation results, we selected the entropy-TOPSIS method to measure the level of rural resilience. The Technique for Order Preference by Similarity to an Ideal Solution (TOPSIS) was used to study the distance between the evaluation object and the ideal solution to obtain the final closeness degree [50]. The core of the entropy-TOPSIS method lies in TOPSIS, but in determining the weights, the entropy weight method was chosen for the assignment [51]. The entropy weight method is an objective assignment method that can avoid the bias brought by subjective assignment to rural resilience [52]. It determines the size of information in each indicator according to the degree of difference between the information within the indicators so that the measurement result of rural resilience level is more objective and fairer. In practical situations, evaluation results are subject to uncertainty due to different scholars’ perceptions and preferences. At the same time, there are no uniform criteria for evaluating rural resilience to justify its results. Therefore, in order to verify the stability of the entropy-TOPSIS method, we used the gray evaluation method to validate its results. The specific calculation steps of the entropy-TOPSIS method are as follows:

- Construction of evaluation matrix. Assuming the existence of *m* evaluation indicators and *n* evaluation objects, the original evaluation matrix *Y* for the level of rural resilience is:
  (1) $$Y=\left[\begin{array}{c} \begin{array}{cc} y_{11}\;\;\;y_{12}\;\;\;\cdots & y_{1n}\\ y_{21}\;\;\;y_{22}\;\;\;\cdots & y_{2n} \end{array}\\ \vdots \;\;\;\;\;\;\;\;\;\vdots \;\;\;\;\;\;\;\;\;\vdots \;\;\;\;\;\;\;\;\;\vdots \\ y_{m1}\;\;\;y_{m2}\;\;\;\cdots \;\;\;y_{mn} \end{array}\right]$$
- Data standardization.
  (2) $$\mathrm{Positive}\;\mathrm{indicators}:\;x_{ij}=\frac{y_{ij}-\min\left(y_{ij}\right)}{\max\left(y_{ij}\right)-\min\left(y_{ij}\right)}$$
  (3) $$\mathrm{Negative}\;\mathrm{indicators}:\;x_{ij}=\frac{\max\left(y_{ij}\right)-y_{ij}}{\max\left(y_{ij}\right)-\min\left(y_{ij}\right)}$$
  (4) $$X=\left[\begin{array}{c} \begin{array}{cc} x_{11}\;\;\;x_{12}\;\;\;\cdots & x_{1n}\\ x_{21}\;\;\;x_{22}\;\;\;\cdots & x_{2n} \end{array}\\ \vdots \;\;\;\;\;\;\;\;\;\vdots \;\;\;\;\;\;\;\;\;\vdots \;\;\;\;\;\;\;\;\;\vdots \\ x_{m1}\;\;\;x_{m2}\;\;\;\cdots \;\;\;x_{mn} \end{array}\right]$$
  where X represents the standardized matrix;
- Indicator weights. We used the entropy weight method to determine the weights of the indicators to avoid the possibility of human-caused bias.
  (5) $$e_{i}=-\frac{\sum_{1}^{n}p_{ij}\cdot \ln p_{ij}}{\ln n}$$
  (6) $$w_{i}=\frac{1-e_{i}}{\sum_{1}^{m}\left(1-e_{i}\right)}$$
  where $e_{i}$ represents the entropy value of the ith indicator. $p_{ij}=\frac{x_{ij}}{\sum_{1}^{n}x_{ij}}$ represents the calculation of the weight of the *i*th indicator in year *j*. $w_{i}$ represents the weight of the *i*th indicator. *i* = 1, 2, …, *m*, *m* represents the number of evaluation indicators. *j* = 1, 2, …, *n*, *n* represent the number of evaluation objects;
- Weighted Evaluation Matrix. The weighted evaluation matrix (R) is obtained by combining the standardized matrix (X) with the weights of each indicator ($w_{i}$).
  (7) $$R=[\begin{array}{c} \begin{array}{cc} x_{11}w_{1}\;\;\;x_{12}w_{1}\;\;\;\cdots & x_{1n}w_{1}\\ x_{21}w_{2}\;\;\;x_{22}w_{2}\;\;\;\cdots & x_{2n}w_{2} \end{array}\\ \vdots \;\;\;\;\;\;\;\;\;\vdots \;\;\;\;\;\;\;\;\;\vdots \;\;\;\;\;\;\;\;\;\vdots \\ x_{m1}w_{m}\;\;\;x_{m2}w_{m}\;\;\;\cdots \;\;\;x_{mn}w_{m} \end{array}]=\left[\begin{array}{c} \begin{array}{cc} r_{11}\;\;\;r_{12}\;\;\;\cdots & r_{1n}\\ r_{21}\;\;\;r_{22}\;\;\;\cdots & r_{2n} \end{array}\\ \vdots \;\;\;\;\;\;\;\;\;\vdots \;\;\;\;\;\;\;\;\;\vdots \;\;\;\;\;\;\;\;\;\vdots \\ r_{m1}\;\;\;r_{m2}\;\;\;\cdots \;\;\;r_{mn} \end{array}\right]$$
- Positive and negative ideal solutions.
  (8) $$R^{+}=\left\{\underset{1\leq i\leq m}{\max}R_{ij}|i=1,2,\cdots ,m\right\}=\left\{R_{1}^{+},R_{2}^{+},\cdots ,R_{m}^{+}\right\}$$
  (9) $$R^{-}=\left\{\underset{1\leq i\leq m}{\max}R_{ij}|i=1,2,\cdots ,m\right\}=\left\{R_{1}^{-},R_{2}^{-},\cdots ,R_{m}^{-}\right\}$$
  where $R^{+}$ represents the positive-ideal solution, and $R^{-}$ represents the negative-ideal solution;
- Euclidean distance.
  (10) $$D_{j}^{+}=\sqrt{\overset{m}{\underset{i=1}{\sum }}{\left(R_{ij}-R_{i}^{+}\right)}^{2}}$$
  (11) $$D_{j}^{-}=\sqrt{\overset{m}{\underset{i=1}{\sum }}{\left(R_{ij}-R_{i}^{-}\right)}^{2}}$$
  where $D_{j}^{+}$ represents the Euclidean distance between different evaluation objects and the positive ideal solution. $D_{j}^{-}$ represents the Euclidean distance between different evaluation objects and the negative ideal solution;
- Closeness.
  (12) $$C_{j}=\frac{D_{j}^{-}}{D_{j}^{+}+D_{j}^{-}}\;$$
  where the value range is [0, 1]. The larger the value of $C_{j}$, the closer the rural resilience level of the research object is to the optimal level. $C_{j}=1$ means that the rural resilience level is the highest, and $C_{j}=0$ means that the rural resilience level is the lowest.

#### 2.2.2. Spatial Autocorrelation Model

The spatial autocorrelation model is a common model used to analyze regional spatial correlation and heterogeneity, and the spatial autocorrelation of rural resilience level can be portrayed by Morans’I index. In this study, we first determined the spatial correlation of rural resilience in 31 regions study regions through global spatial autocorrelation and then reflected the spatial clustering of specific regions with neighboring regions through local spatial autocorrelation [53,54]. The global spatial autocorrelation is measured by GlobalMorans’ I, and its value range is [−1.0, 1.0]. When Morans’ I>0, it indicates that rural resilience has a positive spatial correlation, and the larger the value, the more obvious the correlation. When Morans’ I<0, it indicates that rural resilience has a negative spatial correlation, and the smaller the value, the more obvious the difference. When Morans’ I=0, it indicates that rural resilience is randomly distributed in space. Local spatial autocorrelation is measured by LocalMorans’ I to find the spatial differences caused by the spatial correlation of rural resilience, and to determine the spatially high-incidence areas of rural resilience, so as to compensate for the shortage of global spatial autocorrelation. Local spatial autocorrelation can form five types of spatial distribution characteristics: high–high cluster, high–low cluster, low–high cluster, low–low cluster and not significant.

#### 2.2.3. Qualitative Comparative Analysis Method (QCA)

The evaluation of rural resilience focuses on the complexity and regional characteristics of the rural regional system, and its resilience level is the result of the combination of several condition variables. Qualitative comparative analysis (QCA) can simplify the relationship between condition variables and outcome variables and effectively and systematically deal with the research data of multi-case comparison [55]. It can mine the complex nonlinear relationship between multiple factors [56]. QCA, originally proposed by Charles Ragin, follows Mill’s comparative approach and uses Boolean algebraic logic to make causal inferences [57]. Additionally, it is a case-study-oriented theoretical pooling research method that combines the respective strengths of qualitative and quantitative analysis and helps to answer multiple concurrent causal relationships. QCA can be classified as a clear set qualitative comparative analysis (csQCA), multi-valued qualitative comparative analysis (mvQCA) and fuzzy set qualitative comparative analysis (fsQCA) according to the type of variables [58]. Considering that fsQCA is more advantageous in calibrating fixed distance or fixed ratio variables [59] and can improve the accuracy of the data [60], we selected fsQCA to conduct a group analysis on the drivers of rural resilience level. Therefore, we could solve several “group configurations” [61] and summarize several paths with equivalent results [45] to find suitable paths for rural resilience level improvement. In this study, the outcome variable is the level of rural resilience in 2019, and the conditional variables are administrative force, market force, labor force, technology force and cultural force. FsQCA was used to explore the condition configurations and paths that affect the level of rural resilience. The specific operation steps are calibration variables; variable recalibration and set membership score; necessity analysis; selection of case frequency and threshold; generation of the truth table; identification of core conditions and edge conditions through counterfactual analysis and solution, reporting fsQCA results with symbols; and finally, interpretation of results.

#### 2.2.4. Data Sources

China has always attached importance to rural construction [62]. Therefore, we took 2010 as the starting point to measure the resilience level of the countryside in the consecutive decade of 2010–2019. By considering the ongoing impact of the COVID-19 pandemic and the unavailability of relevant data, the year 2020 and later years were not included in this study for the time being. We took 31 provinces, autonomous regions and municipalities directly under the central government (excluding Hong Kong, Macao and Taiwan) (referred to as 31 regions) as the research objects. Additionally, we studied the regional development degree, dynamic attitude and difference degree situation of rural resilience level under provincial scale so as to provide decision reference and data support for improving rural resilience level. This study is mainly based on the *China Rural Statistical Yearbook* (2011–2020), *China Statistical Yearbook* (2011–2020), *China Social Statistical Yearbook* (2011–2020), *China Urban and Rural Construction Statistical Yearbook* (2011–2020), *China Environmental Statistical Yearbook* (2011–2020), and the statistical yearbooks of each province (2011–2020) and the *Statistical Bulletin of National Economic and Social Development* (2010–2019) to obtain the relevant raw data. The missing data were processed by interpolation method to make up the data.

## 3. Results

### 3.1. Analysis of the Spatial and Temporal Evolution of Rural Resilience Levels

We applied the entropy-TOPSIS method to comprehensively evaluate the rural resilience levels of 31 regions nationwide from 2010 to 2019. Firstly, the weights of each indicator were calculated according to Equations (1)–(6) (Table 1), and secondly, the closeness was calculated according to Equations (7)–(12); that is, the value of rural resilience level. Finally, the grey evaluation method was used to test the robustness of the results of the entropy-TOPSIS method. The entropy-TOPSIS method and grey evaluation method were compared and analyzed, and the ranking results are shown in Table 3. Among them, Jiangsu, Shanghai, Shandong and Beijing ranked in the top four under the entropy-TOPSIS method, while Shanghai, Jiangsu, Beijing and Tianjin were ranked by the grey evaluation method. We can see that the top four results are basically the same. Similarly, the bottom four results are Tibet, Inner Mongolia, Gansu, Qinghai and Gansu, Qinghai, Ningxia and Inner Mongolia. We can see that the results of the bottom four are not significantly changed. The ranking of Henan, Yunnan and Xinjiang remained consistent under the two methods. There was no significant difference in the final ranking obtained by the two methods, and the overall ranking trend was consistent, indicating that the results obtained by the entropy-TOPSIS method have a certain validity. Therefore, on the basis of verifying the validity of the results, we further analyzed the results of the entropy-TOPSIS method.

According to the measurement results of rural resilience level (Table 4), the overall rural resilience level of 31 regions is low, and the national average value is only 0.410 in 2019, of which the highest value is 0.622 in Jiangsu Province, and only Jiangsu Province has a value of more than 0.6. However, from the perspective of time series, the national average level increased from 0.264 in 2010 to 0.410 in 2019, an increase of 55.30%. 

#### 3.1.1. Time Evolution Characteristics

From the dynamic evolution over time, the overall trend of rural resilience level in China’s provinces and municipalities from 2010 to 2019 is rising faster in the eastern region than in the central and western regions. Specifically, the development of China’s rural resilience level formed three stages. 

From 2010 to 2013, China’s rural resilience level showed fluctuating changes, among which Beijing, Shanghai, Hainan and Gansu showed an “N” type rising trend; Qinghai showed a slow downward trend; and other areas showed a slow upward trend. In 2012, due to the continuous fermentation of the European debt crisis and the dry climate in some major grain-producing countries, the international agricultural product market and price fluctuated violently. Although the domestic bulk market operated smoothly, the price fluctuated wildly, leading to a great impact on Beijing, Shanghai and other places.

From 2014 to 2016, China’s rural resilience level is in the stage of adjustment and improvement; 93.55% of the regions are showing a continuous rise, among which Shandong, Shanghai and Jiangsu rural resilience levels exceeded 0.500 for the first time during this period. Guizhou, Beijing and Hunan had the largest increase in rural resilience in 2015, with 24.75%, 12.41% and 11.64%, respectively. Fujian, Qinghai, Jiangxi and Guangdong had larger increases in rural resilience levels of 18.30%, 17.92%, 13.65% and 12.28%, respectively, in 2016. It reflects that the infrastructure construction in key areas of rural areas in the middle and late period of the 18th National Congress of the Communist Party of China (CPC) continues to advance orderly, rural public services and rural governance are gradually improved, and the division of labor and structure of rural industries are gradually rationalized, and the level of rural resilience is accelerated at this stage.

From 2017 to 2019, China’s rural resilience level showed a rapid rise, and the rural resilience level grew most rapidly in this period. Among these, the rural resilience level in Shaanxi increased from 0.306 to 0.418 in 2018 and 2019, an increase of 36.60%. The rural resilience level in Liaoning increased from 0.326 to 0.421 in 2017 and 2018, an increase of 29.14%. It indicates that after long-term and systematic resilience construction, the ability of the countryside to effectively withstand disasters and risks increased. Additionally, the concept of resilience construction is emphasized in the 19th National Congress report, which promotes the comprehensive construction of a rural resilience framework and promotes the high-quality and sustainable development of rural construction.

#### 3.1.2. Spatial Distribution Characteristics

In terms of the overall spatial distribution characteristics, there is a large gap in the level of resilience within the 31 provinces and urban areas, and there is a clear differentiation. Among them, the overall level of rural resilience in the eastern region is high, and only Hebei (0.401) is lower than the national average (0.410) in 2019. While the overall level of rural resilience in the central and western regions is low, especially among the 10 provinces and municipalities in the western region, only Sichuan (0.431) and Shaanxi (0.418) are higher than the national average in 2019, and Qinghai is the lowest (0.236). In order to further portray the spatial distribution characteristics of rural resilience level, Moran’s index (Moran’s I) was chosen to measure the correlation and agglomeration of spatial distribution and analyze whether the national rural resilience level has spatial autocorrelation with the help of ArcGIS10.7 software.

The global spatial autocorrelation analysis was first conducted to calculate the correlation of the rural resilience data through the global Moran index, and the results of the global Moran index are shown in Table 5. The Moran’s I value for rural resilience was all positive, between 0.535 and 0.665, and the *p*-values for the correlation tests during the study period were 0.000 (less than 0.01), indicating that all years of rural resilience significantly rejected the original hypothesis at the 1% level. It indicates that the rural resilience levels of 31 regions in China from 2010 to 2019 are not randomly distributed but show spatial clustering between similar values, showing a strong spatial dependence. Additionally, the areas with high values of rural toughness levels tend to be centrally distributed, and the areas with low values also tend to be centrally distributed, generally showing H-H and L-L clusters, while H-L and L-H clusters are relatively rare.

**Figure 2:**
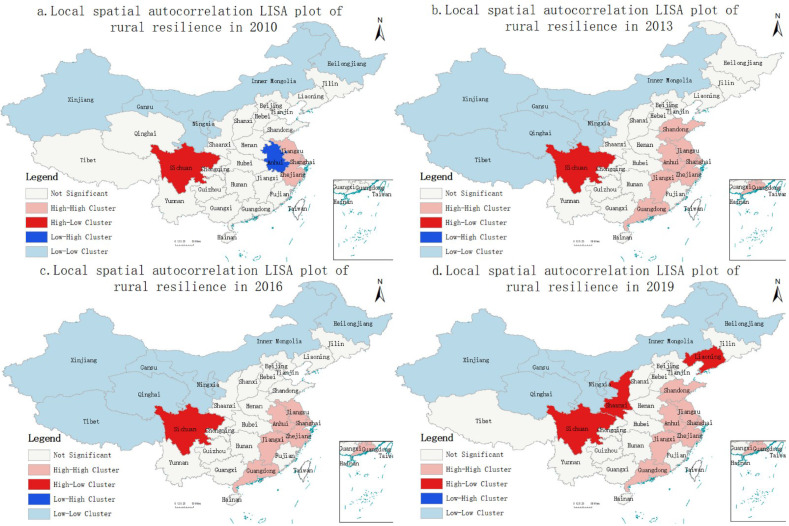
Local spatial autocorrelation LISA plots for rural resilience, 2010, 2013, 2016, 2019.

After passing the global autocorrelation test, the local autocorrelation analysis was conducted, and the local spatial autocorrelation LISA maps of rural resilience in 2010, 2013, 2016 and 2019 were plotted by ArcGIS 10.7 software (Figure 2), and it was found that the H-H, L-L and H-L clusters showed an expanding trend, while the L-H cluster gradually disappeared. This further indicates that the horizontal polarization effect of rural resilience in China is significant, and the spatial distribution is unbalanced. 

H-H cluster is mainly distributed in the eastern region of China, with a stable expansion trend and obvious spatial spillover effects. In 2010, only three regions, Jiangsu, Shanghai and Zhejiang, were located in the H-H cluster region. Additionally, in 2019, Shandong, Jiangxi and Guangdong all transformed from not significant to H-H cluster, and Anhui transformed from L-H cluster to H-H cluster. It shows that Jiangsu, Shanghai and Zhejiang have significant polarization effects, and have a strong radiative driving effect on the surrounding areas, leading Anhui, Shandong, Jiangxi and Guangdong to transform to a high resilience level and finally form an H-H cluster region led by the Yangtze River Delta urban agglomeration and coordinated development of the eastern region.

H-L clusters are mainly distributed on the east side of the Hu Huanyong Line (Appendix A, Item 3), and the number of regions gradually increases. The Sichuan region is always in the H-L cluster during the study period, and the surrounding areas such as Tibet, Qinghai, Gansu and Chongqing generally have lower levels of rural resilience. Therefore, among the western regions, Sichuan shows the phenomenon of leading developers in the west by virtue of its superior agricultural and water resources conditions, its advantageous geographical location along the Belt and Road and Yangtze River Economic Belt and the support of national strategies such as the Western Development and the Chengdu–Chongqing economic circle. Shaanxi and Liaoning transformed from not significant to H-L clusters in 2019, and their rural resilience levels were 0.418 and 0.437, respectively, with obvious enhancement effects and rapidly widening the gap with the surrounding areas.

The L-H cluster had the least number of distribution areas; only Anhui was located in the L-H cluster in 2010, with low rural resilience levels. While the surrounding areas such as Jiangsu, Shanghai and Zhejiang had high rural resilience levels, and Anhui was subject to the siphoning effect of surrounding high-level areas, Anhui’s rural labor, capital and technology and other factors flow out to Shanghai, Jiangsu and Zhejiang regions, making Anhui fall into the marginalization of development, thus limiting the improvement of Anhui’s rural resilience level.

The L-L cluster is mainly distributed in the area west of Hu Line, covering the largest area. Among which Xinjiang, Gansu, Ningxia and Inner Mongolia are always located in the L-L cluster of the 2010–2019 period, Qinghai enters the L-L cluster from not significant, while Tibet and Heilongjiang repeatedly change between not significant and L-L cluster, but the overall development pattern does not change significantly. The not significant regions are mainly concentrated in the central region, which is a largely agricultural province gathering area and generally attaches importance to agricultural development, promoting more development factors to cluster in the rural areas, and its rural resilience level is always at a medium level. The mean values of rural resilience in Hebei, Henan, Hubei, Hunan and Guangxi are 0.327, 0.400, 0.372, 0.349 and 0.326, respectively, so there is no significant cluster in spatial distribution.

### 3.2. Configuration Analysis of the Influencing Factors of Rural Resilience

#### 3.2.1. Data Calibration and Necessity Analysis

Calibration is the process of transforming data into sets and assigning a set membership score to each case [63]. In a qualitative comparative analysis, data calibration should be carried out first. Uncalibrated original data cannot meet the Boolean logic of qualitative comparative analysis [64]. According to the theoretical and practical external knowledge, three qualitative anchors were assigned, which were 95% (full membership), 50% (crossover) and 5% (full non-membership) of the sample data [65], and the calibrated ensemble data fell within the interval [0, 1] (Table 6).

Secondly, in line with mainstream qualitative comparative analysis studies, we tested whether single condition variables (including non-sets) constitute a necessary condition for rural resilience through fsQCA 3.0 software. Necessity analysis means exploring the extent to which the set of outcomes constitutes a subset of the set of conditions [66], that is, without the presence of the condition directly causing the outcome not to occur [67]. An important indicator for judging the conditional variables to be necessary is consistency, which is identified as a necessary condition when the consistency score is greater than 0.9 [5], and the necessary condition tests for high and non-high levels of rural resilience are shown in Table 6. The consistency of Table 7 shows that the consistency score of the single condition is less than 0.9 for both high rural resilience and non-high rural resilience, so the single condition has a limited explanation for the outcome variable and cannot constitute a necessary condition for rural resilience alone.

fsQCA, from the perspective of configuration and based on inductive logic, focuses on analyzing the combination of multiple conditions leading to a particular result [46] and reveals the complex causal relationship through different types of counterfactual analysis [68]. Depending on whether and what kind of counterfactual analysis is performed, fsQCA can obtain three solutions: complex solution (without counterfactual analysis), intermediate solution (based on simple class counterfactual analysis) and parsimony solution (based on simple class and complex class counterfactual analysis), among which the condition variables that appear in both intermediate solution and parsimony solution are called core conditions, and the condition variables that only appear in intermediate solution are called marginal conditions [68]. 

In this study, the data of each provincial and municipality were analyzed by fsQCA 3.0 software, and due to the small sample size, the frequency was set to 1, the threshold was 0.8 [68] and the PRI value was 0.75 [59], and four configurations of high-level rural resilience (H1–H4) and three configurations of non-high level rural resilience (NH1–NH3) were obtained after the operation. As shown in Table 8, the overall coverage of high-level and non-high-level rural toughness is 0.7979 and 0.6775, respectively, indicating that the histological configuration has good explanatory power for the outcome variables. The overall consistency of high level and non-high level rural resilience is 0.8380 and 0.9134, and the consistency of each histological configuration individually is greater than 0.8, indicating that the solution is meaningful and this operation can as the main reason for explaining the high and low level of rural resilience. In order to avoid the repeatability of cases with similar but different configuration sources [69], we merge the configuration H1 and H2 with the same labor force as the core condition so as to obtain three explanatory paths of high-level rural resilience and three explanatory paths of non-high-level rural resilience.

#### 3.2.2. Analysis of Conditional Configuration Results

1. High-level rural resilience explanation path

Labor driven, containing the configurations H1 (~MAR*LAB*~TEC*CUL) and H2 (~ADM*~MAR*LAB*CUL), has a consistency of 0.8681 and 0.8817 and coverage of 0.4047 and 0.4151, respectively, which means that configurations H1 can explain about 40.47% of the rural resilience cases and configurations H2 can explain about 41.51% of the rural resilience cases. Under this path, regardless of the presence or absence of administrative, market, technology and cultural forces, a high level of rural resilience can be achieved when labor is present as a core condition and plays a driving role, demonstrating that labor is the core influencing factor to enhance rural resilience. Therefore, various measures were taken to promote the full employment of rural labor. For example, Beijing, Jiangsu, Guangdong and Heilongjiang provinces adopted vocational skills training, coordinated urban and rural employment, and provided subsidies and loans to support entrepreneurship. It has strengthened the construction of rural professionals, encouraged social talents to participate in rural construction, promoted the employment of rural labor in nearby areas and across regions and alleviated the problem of rural “hollowing out”. This is a key source of support for the revitalization of talents in the rural revitalization strategy.

Market–labor–technology linkage-driven, containing the configuration H3 (ADM*MAR*LAB*TEC), has a consistency of 0.9061 and coverage of 0.3498, indicating that this path explains about 34.98% of the rural resilience cases. The path has a core presence of market power, labor force and technology force at the same time and shows higher consistency for the outcome variables, showing that rural resilience is more likely to perform at high levels in regions where these three influences act in conjunction. For example, Hunan, Hebei, Henan, Guangxi and other regions, supported by policies to strengthen agriculture, benefit farmers and enrich farmers, and rely on the industrial advantages of major agricultural provinces, have continuously increased their comprehensive agricultural production capacity and formed new business forms such as the “agriculture+” model. It has helped the revitalization of the industry; realized the deep integration of agriculture, rural areas and farmers; radiated and revitalized various advantages of rural resources; enhanced the internal driving force of rural areas; and improved the ability of rural areas to cope with and resolve risks.

Cultural driven, containing the configuration H4 (MAR*~LAB*~TEC*CUL), has a consistency of 0.9130 and coverage of 0.3050, manifesting that this pathway explains about 30.50% of rural resilience cases. Cultural force plays a core role in this path, market force is shown to exist as a marginal condition, and labor and technology forces are shown to be absent as marginal conditions, indicating that cultural force plays a key role in such paths. The rural area is the basic carrier of Chinese culture and rural culture, as an important spiritual force, has a significant impact on rural production and lifestyle and is an important guarantee for the realization of rural revitalization. For example, Fujian insists on the integration of culture and tourism, relying on the profound Hakka culture, continuously tapping rural cultural and tourism resources, highlighting the characteristics of cultural and tourism integration and helping rural industry and tourism to develop in-depth through cultural construction. Since 2002, Zhejiang began to attach importance to the construction of a beautiful village, the development and market operation of regional cultural resources have been carried out, a large number of rural scenic spots have been promoted and the leap development of rural public cultural products and services driven by collective economy has been realized, thus laying a solid foundation for strengthening rural resilience.

2. Non-high level rural resilience explanation path

Market–labor absent, which contains the configuration NH1 (~ADM*~MAR* ~LAB*~*CUL*), has a consistency of 0.9616 and coverage of 0.3541, manifesting that this path can explain about 35.41% of rural resilience cases. When the market force and labor force are absent as core condition in this path, the market dynamics and human capital in the rural resilience system is not conducive to the improvement of resilience level in rural areas. For example, in Gansu and Ningxia, the labor force population showed a downward trend, the industrial structure in Gansu was low-end and heavily industrialized, the market resource allocation was insufficient and the absorption capacity of the labor force was seriously insufficient. In Ningxia, the vicious circle of “labor force loss and market depression” leads to the slow improvement of rural resilience.

Administrative–market absent, which contains the configuration NH2 (~ADM*~MAR* ~LAB*TEC), has a consistency of 0.9304 and coverage of 0.3953, which means that this path explains about 39.53% of rural resilience cases. The absence of administrative and market forces in this path plays a central role, suggesting that even at high levels of science and technology when villages are in a high-shock environment, the level of rural resilience will be suppressed as long as there are not sufficient policy support and market conditions. Chongqing and Jilin, for example, need national policy support and deep rural market cultivation to stimulate rural industries and market dynamics through rural policy dividends in order to raise their rural resilience levels.

Cultural absence, which contains the configuration NH3 (~ADM*~LAB*TEC*~*CUL*), has a consistency of 0.9610 and coverage of 0.3804, which means that this path explains about 38.04% of rural resilience cases. The lack of cultural force plays a central role in this path, indicating that even if technological power exists, as long as a cultural force is absent, it limits the level of rural resilience. This path also proves the culturally driven path of the high-level rural resilience explanation path, further validating the importance of cultural power in improving rural resilience.

## 4. Discussion

According to the rural resilience level measurement and spatiotemporal evolution analysis of 31 research subjects in China from 2010 to 2019, this study found that the overall rural resilience level was low and showed an upward trend. This is consistent with the results demonstrated by Huang and Li et al. [22,38]. Li also found that during the study period, China’s rural resilience level remained below the medium level and showed an upward trend [38]. As can be seen from Table 4, the eastern region has the fastest improvement, while the central and western regions have slower growth rates. Significant achievements were made in building resilience in China’s rural areas, but there is still a lot of room for improvement. In terms of rural construction, China needs to continue to adhere to the rural revitalization strategy. On the basis of following the actual situation in rural areas, China should enhance the development vitality of agriculture, rural areas and farmers and further improve the level of rural resilience. First of all, we paid attention to the great contribution brought by the rural revitalization strategy. As a policy tool, the rural revitalization strategy is an important factor affecting the level of rural resilience. However, the implementation of the current rural revitalization strategy is mainly based on the “Rural Revitalization Strategic Plan (2018–2022)” as the construction program, resulting in the phenomenon that all regions are basically built according to the same standard. However, homogeneous policy supply leads to policy failure, resulting in deviations or inefficiencies in the practice of rural revitalization, thereby affecting the improvement of rural resilience.

The distribution of rural resilience in China shows obvious spatial aggregation, and the overall spatial distribution is uneven. This is consistent with the findings of Su and Luo [70]. Among them, H-H, L-L and H-L Cluster tended to expand, while L-H Cluster gradually disappeared. Su and Chang distinguished differences in resilience between urban and rural areas through spatial autocorrelation analysis of resilience indicators, and local autocorrelation results for each indicator varied [71]. The idea of establishing an index system in this study is also based on urban–rural differences, which confirms Su’s research [71]. Based on the characteristics of uneven development of rural resilience, we believe that various regions should take differentiated measures to achieve the goal of promoting balanced regional development in the future.

This study used the fsQCA method to analyze rural resilience in 31 regions of China. There are differences in the key factors and combinations that affect the level of rural resilience in different regions. Therefore, all regions should pay attention to the key influencing factors and their configurations and choose the improvement path according to local conditions. This further confirms Li’s point [19]. Li believes that rural areas should formulate rural development strategies based on the dominant factors and their characteristics [19]. The results of the study show that labor and cultural forces are the only key factors to effectively improving the level of rural resilience. In his research, Li pointed out that China’s traditional agricultural labor shortage and industrial recession are likely to lead to low levels of resilience [38]. The combination of market, labor and technology forces can effectively improve the level of rural resilience. 

The indicator system and research framework of this study are innovative (Figure 1). However, it is difficult to obtain some indicator data in China’s rural areas. Therefore, there are limitations in the selection of indicators. In addition, the resilience level and spatial analysis in this paper are temporally dynamic, and the research on influencing factors only selects 2019 as the analysis object. The fsQCA method used in this study lacked the consideration of time effects. Future research should try to introduce the time dimension into the analysis of fsQCA. The rural revitalization strategy is a new path for China’s rural construction. Since China’s rural policies have a great impact on rural development, we combine China’s rural resilience with the rural revitalization strategy. We emphasized rural resilience as distinct from urban resilience. Urban resilience focuses on the construction of economic, social, ecological and infrastructure resilience. We believe that rural areas should pay more attention to industrial resilience, ecological resilience, cultural resilience, organizational resilience and livelihood resilience. By revealing the way of China’s rural construction, it will bring reference and enlightenment to other countries. We encourage other countries to conduct localized rural construction in light of their national conditions, which is conducive to sustainable rural development.

## 5. Conclusions

This paper takes 31 regions in China (excluding Hong Kong, Macao and Taiwan) as the research area and case samples and uses the entropy weight-TOPSIS method and spatial autocorrelation to measure their rural resilience levels and spatial distribution characteristics from 2010 to 2019. Then we explored the configurations and paths that affect the level of rural resilience through fsQCA.

During the study period, the average level of Chinese rural resilience increased from 0.264 in 2010 to 0.410 in 2019, an increase of 55.30%. It shows that the development trend of rural resilience is good, and the overall trend is stable. Especially after the rural revitalization strategy was put forward, the increasing trend and stability of the resilience level of China’s rural areas became more obvious. Therefore, China should pay attention to innovation in the regional implementation of the rural revitalization strategy. On the one hand, it is necessary to give full play to the leading role of the government. The promotion of rural resilience and rural revitalization is planned as a whole by forming a policy cluster consisting of multi-level policies. On the other hand, on the basis of the guiding direction of the rural revitalization strategy, each region should explore and innovate in practice to form a preset framework and strategic institutional supply.

The level of rural resilience has a strong spatial dependence, showing a significant positive spatial correlation. With the passage of time, the agglomeration distribution characteristics of regions with similar levels of resilience have become increasingly uneven and have shown an expanding trend. H-H is generally distributed in the eastern region. L-L is distributed on the west side of the Hu Huanyong Line. The insignificant regions are mainly located in the central region. The H-L appears at the junction of the eastern, central and western regions. The high-value agglomeration areas in the eastern region, as well as Sichuan, Shaanxi and Liaoning, as dominant regions, must maintain their dominant position for a long time and strengthen their radiating and driving effect on the surrounding areas. With the help of the three-sided radiation effect of Zhejiang, Jiangxi and Guangdong, Fujian improves rural resilience through ecological transformation and utilization of renewable energy. Beijing, Tianjin and Jilin should strengthen the promotion of the agricultural mechanization rate and improve the construction of agricultural industry technology systems so as to improve the ability of rural areas to deal with shocks and risks. The central region relies on the leading role of the high-value agglomeration areas in the eastern region, Sichuan and Shaanxi. The central region should actively adjust the agricultural and industrial structure in the middle zone and give full play to the advantages of the factor markets and commodity markets in each region. On the one hand, the western region relies on the spatial spillover effects of Sichuan and Shaanxi, and on the other hand, it should establish a deep cooperative relationship with the eastern region.

The consistency of each antecedent condition does not exceed 0.9, which does not constitute a necessary condition, indicating that rural resilience is not the result of the independent influence of a single factor, but there are multiple paths for improvement or obstruction. There are three driving paths that affect the level of rural resilience, namely, labor-driven, market–labor–technology linkage-driven and cultural-driven. There are three obstacle paths affecting the resilience level of villages, namely, market–labor absent, administrative–market absent and cultural absent. The original coverage of each route has a small difference, and the impact on rural resilience is similar. Therefore, we suggest that regions should jointly improve the allocation level of these three influencing factors through the active guidance of the government and the market. For example, the eastern region should give full play to its advantages in labor and cultural strength. Based on the existing advantages of the agricultural industry, the central region makes concerted efforts in finance, market, employment and technology. The western region should start from a non-high perspective and find the key factors and paths that hinder the improvement of rural resilience. The western region can solve the problem of rural resilience development by strengthening the connection with the surrounding neighboring regions and the eastern region.

## Figures and Tables

**Table 1:** Rural resilience evaluation index system.

Target	Dimensions	Indicators ^1^	Properties ^2^	Weights
Rural Resilience	Industrial resilience (0.157)	Added value of agriculture, forestry, animal husbandry and fishery industries	+	0.116
		Output value of total regional agricultural	+	0.004
		Added value of the secondary and tertiary industries/GDP of the year	+	0.021
		Proportion of rural 16–64 years old to the total population	+	0.016
	Ecological resilience (0.266)	Rural greening coverage rate	+	0.088
		Drainage culvert density	+	0.084
		Fertilizer application intensity per unit of arable land area	−	0.030
		Prevalence rate of harmless sanitary toilets in rural areas	+	0.064
	Cultural resilience (0.212)	Number of national civilized villages and towns	+	0.099
		Proportion of people with high school education or above to the total number of people	+	0.052
		Proportion of rural grassroots organization personnel to the total rural population	+	0.039
		Average number of cultural stations per township	+	0.022
	Organizational resilience (0.221)	Proportion of financial expenditures on employment and health care to general public expenditures	+	0.035
		Proportion of public security expenditures to general public expenditures	+	0.047
		Area of roads per capita	+	0.041
		Household access rate of rural cable radio and TV	+	0.098
	Livelihood resilience (0.144)	Rural residents’ savings rate	+	0.017
		Rural residents’ per capita disposable income	+	0.076
		Rural population employment rate	+	0.030
		Rural retail sales growth rate of consumer goods	+	0.021

Note: ^1^ The interpretation of indicators can be found in Appendix A Table A1. ^2^ In the properties, + represents a positive indicator; the larger the indicator value, the better the evaluation. − represents a negative indicator; the smaller the indicator value, the better the evaluation.

**Table 2:** Variable selection and definition.

Antecedent Condition		Variable Selection	Variable Definition
Administrative force	ADM	Per capita finance expenditure of agriculture, forestry and water resources	The attention and support of governments at all levels to rural construction
Market force	MAR	Rural construction input	The effective allocation ability of rural market resources
Labor force	LAB	Years of schooling per capita	The ability of talent to support rural construction
Technology force	TEC	Level of agricultural machinery	The ability of rural industries, especially agricultural science and technology innovation
Cultural force	CUL	Per capita cultural and entertainment consumption expenditure	The identity and cohesiveness of the countryside based on culture

**Table 3:** Ranking and comparison of evaluation levels of rural resilience.

Region	Entropy-TOPSIS Method		Gray Evaluation Method		Ranking Change Results
	Results	Ranking	Results	Ranking	
Beijing	0.445	4	0.415	3	1
Tianjin	0.380	9	0.408	4	5
Hebei	0.327	15	0.371	7	8
Liaoning	0.315	18	0.354	10	8
Shanghai	0.495	2	0.498	1	1
Jiangsu	0.520	1	0.483	2	1
Zhejiang	0.431	5	0.373	6	1
Fujian	0.419	7	0.363	9	2
Shandong	0.466	3	0.402	5	2
Guangdong	0.428	6	0.346	11	5
Hainan	0.370	11	0.339	12	1
Shanxi	0.282	20	0.335	13	7
Inner Mongolia	0.220	29	0.225	31	2
Jilin	0.237	27	0.270	26	1
Heilongjiang	0.279	21	0.316	14	7
Anhui	0.319	17	0.312	15	2
Jiangxi	0.338	14	0.311	17	3
Henan	0.400	8	0.367	8	0
Hubei	0.372	10	0.304	18	8
Hunan	0.349	12	0.311	16	4
Guangxi	0.325	16	0.296	20	4
Chongqing	0.278	22	0.290	21	1
Sichuan	0.345	13	0.286	22	9
Guizhou	0.245	25	0.281	23	2
Yunnan	0.255	24	0.277	24	0
Tibet	0.229	28	0.271	25	3
Shaanxi	0.267	23	0.264	27	4
Gansu	0.204	30	0.257	28	2
Qinghai	0.188	31	0.251	29	2
Ningxia	0.239	26	0.231	30	4
Xinjiang	0.284	19	0.299	19	0

**Table 4:** Rural resilience level values by region, 2010–2019.

Area	Region	2010	2011	2012	2013	2014	2015	2016	2017	2018	2019
Eastern region	Beijing	0.392	0.411	0.387	0.416	0.403	0.453	0.482	0.486	0.494	0.530
	Tianjin	0.305	0.320	0.333	0.351	0.366	0.384	0.422	0.422	0.442	0.462
	Hebei	0.259	0.279	0.296	0.312	0.325	0.337	0.356	0.348	0.357	0.401
	Liaoning	0.237	0.251	0.271	0.275	0.290	0.314	0.330	0.326	0.421	0.437
	Shanghai	0.460	0.477	0.425	0.493	0.497	0.500	0.510	0.519	0.528	0.549
	Jiangsu	0.393	0.440	0.477	0.491	0.515	0.545	0.562	0.583	0.573	0.622
	Zhejiang	0.330	0.358	0.376	0.403	0.413	0.436	0.473	0.484	0.511	0.529
	Fujian	0.331	0.374	0.382	0.403	0.420	0.388	0.459	0.454	0.479	0.508
	Shandong	0.377	0.411	0.436	0.445	0.449	0.477	0.511	0.510	0.519	0.534
	Guangdong	0.356	0.388	0.385	0.413	0.400	0.399	0.448	0.482	0.493	0.524
	Hainan	0.265	0.320	0.301	0.360	0.364	0.380	0.395	0.422	0.444	0.452
Central region	Shanxi	0.245	0.248	0.261	0.266	0.279	0.290	0.306	0.299	0.303	0.326
	Inner Mongolia	0.183	0.194	0.204	0.208	0.209	0.217	0.239	0.249	0.246	0.259
	Jilin	0.194	0.208	0.213	0.215	0.232	0.247	0.267	0.248	0.260	0.293
	Heilongjiang	0.217	0.229	0.253	0.272	0.270	0.287	0.299	0.310	0.322	0.336
	Anhui	0.234	0.253	0.268	0.302	0.315	0.336	0.353	0.362	0.368	0.400
	Jiangxi	0.244	0.279	0.299	0.315	0.318	0.337	0.383	0.398	0.404	0.408
	Henan	0.346	0.363	0.369	0.386	0.404	0.414	0.426	0.402	0.432	0.463
	Hubei	0.286	0.316	0.333	0.343	0.348	0.384	0.421	0.415	0.422	0.454
	Hunan	0.271	0.289	0.295	0.315	0.335	0.374	0.394	0.385	0.392	0.443
	Guangxi	0.247	0.263	0.275	0.300	0.312	0.342	0.355	0.359	0.385	0.418
Western region	Chongqing	0.221	0.237	0.248	0.251	0.262	0.282	0.309	0.304	0.320	0.354
	Sichuan	0.268	0.294	0.312	0.321	0.329	0.345	0.363	0.390	0.405	0.431
	Guizhou	0.167	0.180	0.190	0.181	0.198	0.247	0.274	0.302	0.341	0.374
	Yunnan	0.188	0.198	0.210	0.226	0.236	0.256	0.279	0.293	0.311	0.356
	Tibet	0.200	0.206	0.211	0.207	0.215	0.221	0.238	0.246	0.275	0.275
	Shaanxi	0.210	0.219	0.225	0.237	0.244	0.262	0.282	0.267	0.306	0.418
	Gansu	0.172	0.187	0.176	0.189	0.193	0.203	0.225	0.221	0.231	0.250
	Qinghai	0.182	0.166	0.163	0.162	0.161	0.173	0.204	0.215	0.225	0.236
	Ningxia	0.194	0.205	0.218	0.223	0.226	0.236	0.260	0.272	0.272	0.288
	Xinjiang	0.226	0.233	0.239	0.258	0.271	0.274	0.291	0.316	0.357	0.379
	Average value	0.264	0.284	0.291	0.308	0.316	0.333	0.358	0.364	0.382	0.410

**Table 5:** Global Moran Index of Rural Resilience Levels.

	2010	2011	2012	2013	2014	2015	2016	2017	2018	2019
Moran’s I	0.535 ***	0.557 ***	0.585 ***	0.608 ***	0.628 ***	0.643 ***	0.665 ***	0.634 ***	0.575 ***	0.557 ***
z-score	4.819	4.971	5.204	5.383	5.57	5.703	5.85	5.602	5.092	4.967
*p*-value	0.000	0.000	0.000	0.000	0.000	0.000	0.000	0.000	0.000	0.000

Note: *** represents significance at the 1% level.

**Table 6:** Calibration table of conditions and result data.

Condition and Result			Calibration		
			Full Membership	Crossover	Full Non-Membership
Condition	Administrative force	ADM	1096.59	736.27	234.78
	Market force	MAR	2.2649	1.4765	0.7837
	Labor force	LAB	586,944.94	170,994.07	2533.055
	Technology force	TEC	8.7598	8.0442	6.7118
	Cultural force	CUL	1829.3077	1423.5187	1049.1165
Result	Rural resilience	RES	0.5418	0.4175	0.2624

**Table 7:** Necessity analysis for high and non-high levels of rural resilience.

Antecedent Condition		High		Non-High	
		Consistency	Coverage	Consistency	Coverage
Administrative force	ADM	0.6826	0.6571	0.6073	0.5892
	~ADM	0.5732	0.5916	0.6725	0.6725
Market force	MAR	0.5635	0.6346	0.5424	0.6156
	~MAR	0.6587	0.5882	0.678	0.6101
Labor force	LAB	0.763	0.7565	0.5437	0.5433
	~LAB	0.5395	0.5399	0.7565	0.7628
Technology force	TEC	0.6289	0.5863	0.7095	0.6666
	~TEC	0.6425	0.687	0.5598	0.6032
Cultural force	CUL	0.7948	0.7523	0.5881	0.5609
	~CUL	0.5362	0.5637	0.7403	0.7842

Note: ~ indicates dispensable.

**Table 8:** Analysis of high-level and non-high-level rural resilience configuration.

Regional Division	High Level				Non-High Level		
Conditional Variables	Labor-Driven		Market–Labor–Technology Linkage-Driven	Cultural-Driven	Market–Labor Absent	Administrative–Market Absent	Cultural Absent
	H1	H2	H3	H4	NH1	NH2	NH3
Administrative force		𐤈	•		𐤈	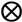	𐤈
Market force	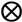	𐤈	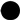	•	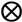	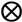	
Labor force	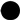	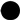	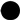	𐤈	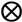	𐤈	𐤈
Technology force	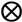		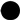	𐤈		•	•
Cultural force	•	•		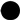	𐤈		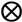
Consistency	0.8681	0.8817	0.9061	0.9130	0.9616	0.9304	0.9610
Raw Coverage	0.4047	0.4151	0.3498	0.3050	0.3541	0.3953	0.3804
Unique Coverage	0.0641	0.0583	0.0084	0.0466	0.0212	0.0316	0.0321
Solution Consistency	0.8380				0.9134		
Solution Coverage	0.7979				0.6775		

Note: 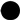 and 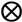 indicate the presence and absence of core variables, • and 𐤈 indicate the presence and absence of marginal conditions, spaces indicate that the condition is optional.

## Data Availability

Not applicable.

## Appendix A

1. The general requirements of rural revitalization strategy is “thriving businesses, pleasant living environments, social etiquette and civility, effective governance, and prosperity”. It puts forward the direction and construction goals of China’s rural development from five aspects: industry, ecology, culture, governance and livelihood;
2. Industry revitalization, talent revitalization, culture revitalization, environment revitalization and organizational structures revitalization are collectively referred to as the five revitalizations. It is the specific requirements of the rural revitalization strategy from five important aspects;
3. Hu Huanyong Line is named after Hu Huanyong, a famous population geographer. Hu Huanyong proposed this line in 1935 to indicate the population density of different areas on either side of the line.

**Table A1:** Interpretation of indicators in the rural resilience evaluation index system.

Dimensions	Indicators	Notes
Industrial resilience	Added value of agriculture, forestry, animal husbandry and fishery industries	To reflect the benefits of local industries
	Output value of total regional agricultural	To reflect the level of local agricultural development
	Added value of the secondary and tertiary industries/GDP of the year	To reflect economic diversification. The secondary industries are mining (excluding ancillary activities), manufacturing (excluding metal products, machinery and equipment repair), production and supply of electricity, heat, gas and water, and construction. The tertiary industry refers to the service industry and refers to industries other than the primary and secondary industries.
	Proportion of rural 16–64 years old to the total population	To reflect the current situation of rural labor force
Ecological resilience	Rural greening coverage rate	To reflect the ecological restoration of rural areas
	Drainage culvert density	To reflect the level of rural ecological environment governance
	Fertilizer application intensity per unit of arable land area	To reflect the impact of rural ecological environment
	Prevalence rate of harmless sanitary toilets in rural areas	To reflect the sustainable development of rural ecological environment. Harmless sanitary toilets refer to toilets that meet the basic requirements of sanitary toilets, have facilities for harmless disposal of feces and are managed according to standards.
Cultural resilience	Number of national civilized villages and towns	To reflect the level of civilization in the region. The national civilized villages and towns in China are evaluated nationwide according to the development of villages and towns in all aspects through the examination and evaluation of organizations and leaders at all levels. The evaluation criteria include five aspects: organization and leadership, creation activities, village appearance, cultural construction, and social fashion.
	Proportion of people with high school education or above to the total number of people	To reflect the level of cultural literacy and education of rural residents
	Proportion of rural grassroots organization personnel to the total rural population	To reflect the civilization level and political literacy of rural residents. The number of rural grassroots organizations here is the number of village committee members.
	Average number of cultural stations per township	To reflect the accessibility of cultural and recreational facilities. Township cultural station is a public welfare institution held by the government. It is a comprehensive public cultural institution that integrates various cultural activities such as reading books and newspapers, publicity and education, literature and entertainment, popular science training, information services, sports and fitness, and serves the local rural masses.
Organizational resilience	Proportion of financial expenditures on employment and health care to general public expenditures	To reflect social security capacity. Employment medical and health expenditure includes social security and employment expenditure and health and health expenditure.
	Proportion of public security expenditures to general public expenditures	To reflect government investment in public security
	Area of roads per capita	To reflect the regional road construction and transportation convenience
	Household access rate of rural cable radio and TV	To reflect the degree of integration in the information age
Livelihood resilience	Rural residents’ savings rate	To reflect the saving capacity of farmers
	Rural residents’ per capita disposable income	To reflect the living standard and rich degree of farmers
	Rural population employment rate	To reflect the employment situation of the rural population and their ability to resist risks
	Rural retail sales growth rate of consumer goods	To reflect the level of rural consumption
